# Narrow *lpa1* Metaxylems Enhance Drought Tolerance and Optimize Water Use for Grain Filling in Dwarf Rice

**DOI:** 10.3389/fpls.2022.894545

**Published:** 2022-05-10

**Authors:** Ryza A. Priatama, Jung Heo, Sung Hoon Kim, Sujeevan Rajendran, Seoa Yoon, Dong-Hoon Jeong, Young-Kug Choo, Jong Hyang Bae, Chul Min Kim, Yeon Hee Lee, Taku Demura, Young Koung Lee, Eun-Young Choi, Chang-deok Han, Soon Ju Park

**Affiliations:** ^1^Division of Applied Life Science (BK21 Program), Plant Molecular Biology and Biotechnology Research Center (PMBBRC), Gyeongsang National University, Jinju, South Korea; ^2^Institute of Plasma Technology, Korea Institute of Fusion Energy, Gunsan, South Korea; ^3^Division of Biological Sciences and Research Institute for Basic Science, Wonkwang University, Iksan, South Korea; ^4^Environmental Exposure & Toxicology Research Center, Korea Institute of Toxicology, Jinju, South Korea; ^5^Department of Horticulture Industry, Wonkwang University, Iksan, South Korea; ^6^Department of Life Science and Multidisciplinary Genome Institute, Hallym University, Chuncheon, South Korea; ^7^National Institute of Agricultural Biotechnology, Suwon, South Korea; ^8^Division of Biological Science, Graduate School of Science and Technology, Nara Institute of Science and Technology, Ikoma, Japan; ^9^Department of Agricultural Science, Korea National Open University, Seoul, South Korea

**Keywords:** metaxylem, drought tolerance, semi-dwarf rice, grain filling, rice

## Abstract

Rice cultivation needs extensive amounts of water. Moreover, increased frequency of droughts and water scarcity has become a global concern for rice cultivation. Hence, optimization of water use is crucial for sustainable agriculture. Here, we characterized *Loose Plant Architecture 1* (*LPA1*) in vasculature development, water transport, drought resistance, and grain yield. We performed genetic combination of *lpa1* with semi-dwarf mutant to offer the optimum rice architecture for more efficient water use. *LPA1* expressed in pre-vascular cells of leaf primordia regulates genes associated with carbohydrate metabolism and cell enlargement. Thus, it plays a role in metaxylem enlargement of the aerial organs. Narrow metaxylem of *lpa1* exhibit leaves curling on sunny day and convey drought tolerance but reduce grain yield in mature plants. However, the genetic combination of *lpa1* with semi-dwarf mutant (*dep1-ko* or *d2*) offer optimal water supply and drought resistance without impacting grain-filling rates. Our results show that water use, and transports can be genetically controlled by optimizing metaxylem vessel size and plant height, which may be utilized for enhancing drought tolerance and offers the potential solution to face the more frequent harsh climate condition in the future.

## Introduction

Rice is a major food crop that is consumed as a staple food by billions of people worldwide (Elert, 2014; OECD/FAO, 2018). During cultivation, rice plants consume extensive amounts of water. Approximately 2,500 l water are estimated to be required for 1 kg rice (Bouman et al., 2007; Linquist et al., 2015). Furthermore, the changing climate is predicted to become a significant challenge in rice cultivation, as water shortages and high temperatures are expected to occur more frequently (Vikram et al., 2015; Korres et al., 2017). Therefore, rice breeding for more efficient water use and stronger drought tolerance is required to overcome these problems. Several strategies to improve plant survival under drought conditions have been proposed, including “water banking,” a method of restricting water use by reducing root hair density and/or xylem diameter and stomatal density (Wasson et al., 2012; Feng et al., 2016; Caine et al., 2019; Mohammed et al., 2019). Previously, a breeding program selecting for smaller xylem vessel areas increased wheat yields by 3–11% under drought conditions (Richards and Passioura, 1989).

In vascular plants, the xylem is responsible for efficient transport of water and minerals over long distances from roots to leaves and also provides mechanical strength (Brodersen et al., 2019). Xylem is categorized into primary and secondary xylem. Primary xylem is differentiated as the cell size increases during the primary cell wall growth stage; secondary cell wall growth promotes the growth of thick walls in secondary xylem of woody plants (Spicer and Groover, 2010; Lucas et al., 2013; Rathgeber et al., 2016). Owing to the absence of secondary xylem in annual plants, protoxylem, and metaxylem vessels are differentiated from the primary xylem. First, protoxylems form annular and spiral vessels, and at a later stage, metaxylems form scalariform and pitted vessels with relatively large lumen diameters (Karam, 2005). The sizes of the xylem vessels, which mainly expand during primary cell wall growth, influence axial water flow in the plant water transport system (i.e., hydraulic conductivity). Hydraulic conductivity depends on vessel characteristics including number, size, and length (Pratt et al., 2021).

Plants can adapt to annual environmental conditions through the regulation of xylem vessel size. In woody plants, annual tree rings play a significant role in plant adaptation to climate (Smith et al., 2013). Narrower xylem vessels are more favorable during droughts and at warm temperatures while larger xylem vessels promote plant growth under water abundant conditions at hot temperatures (Rathgeber et al., 2016). In annual plants, vessel size is determined during the developmental differentiation of the metaxylem and protoxylem. Changes in climatic conditions usually trigger morphological flexibility in root and leaf xylem vessels (Fonti and Jansen, 2012; Myburg et al., 2013; Salazar-Henao et al., 2016).

In a previous report (Park, 2009), *lpa1-2ds* showed a rolling leaf phenotype in the field, which implies that Loose Plant Architecture 1 (LPA1), an INDETERMINATE DOMAIN (IDD) family transcription factor, might be involved in vascular bundle development. *lpa1-1* and *lpa1-2ds* showed wide plant architecture but revertant of *lpa1-2ds* showed normal architecture (Wu et al., 2013; Liu et al., 2016a).

In this study, we characterized the phenotypic expression of *lpa1* and explored breeding strategies to enhance drought tolerance and yield. To understand the role of *LPA1* in metaxylem enlargement, the expression patterns and molecular functions of *LPA1* were explored during vascular development. To evaluate the agricultural utility of *LPA1*-mediated metaxylem enlargement, double genetic combinations of *lpa1* and *dep1* or *d2* were examined for efficiency of water transport, drought tolerance, and grain filling.

## Materials and Methods

### Plant Materials

In this study, we used *lpa1-2ds*, *lpa1-3, lpa1-4*, revertant, *dep1-ko*, and *d2*, which all have *Japonica* rice backgrounds. To generate double mutants, *lpa1-3* was crossed with *dep1-ko* and *d2*, and then backcrossed with *lpa1-3* twice. The progeny of BC2F2 were used for all phenotyping. *lpa1-3* and *lpa1-4* were isolated from the regeneration population of *lpa1-2ds*, as previously reported (Liu et al., 2016a). Briefly, we generated more than 100 regenerated plants for remobilizing *Ds. lpa1-3* and *lpa1-4* alleles were identified by sequencing *Ds* excision sites (Liu et al., 2016a). *lpa1-1*, a *lpa1* allele with an *Indica* rice background, was obtained from Dr. Zhukuan Cheng from the Institute of Genetics and Developmental Biology at the Chinese Academy of Science, Beijing, China.

### Field Growth and Phenotyping of the Rice Plants

To examine the rice plants under natural field conditions, they were grown in the Gyeongsang National University paddy field (Sacheon, South Korea) and Kyungpook National University paddy field (Gunwi, South Korea) from June to October in 2019 and 2020. All the phenotyping data collected from the field in 2020 were used as the representative phenotypes including yield-related traits. The seeds were sterilized overnight at room temperature (25 ± 2°C) using 0.05% fungicide (Samgong spotak; South Korea) and then washed five times. The sterilized seeds were then germinated at 28°C in the dark for 3 days. More than 50 germinated seeds were sowed in seed trays in May and transplanted into the paddy fields in June. The planting density was 15 cm between each plant in a row, with the rows 30 cm apart. Field management, including irrigation, fertilizer application, and pest control, followed normal agricultural practice. Each genotype was represented by more than 20 biological replicates (plants) in the field.

Leaf morphology measurements were carried out using the second leaf from the flag leaf, the largest leaf on the main culm, in mature plants after the flowering stage. Each genotype was represented by a minimum of nine biological replicates (plants) were used for the analyses. The leaves were sealed with clear coating tapes (Scotch^®^ Box Sealing Tape 372, 3 M) before the length (proximal–distal), width, and area of each leaf was measured using an LI-3100 area meter (LI-COR, Lincoln, United States), set to a 1-mm^2^ resolution. For measuring yield-related traits, such as culm height, tiller number, spikelet number per panicle, grain filling rate, and total grain number per panicle, at least 25 mature plants were harvested for each genotype including *lpa1-3*, *dep1-ko*, and *lpa1-3 d2* double mutants in October 2019 and 2020. Damaged or diseased plants were not included in the analyses. After drying for 10 days under natural shaded conditions, the total spikelet number and number of filled grains in each panicle were counted and the grain filling rate was calculated. For examining grain yield, the total grain number per plant was measured by harvesting all the filled grains from each plant.

For the measurement of the total chlorophyll content in the leaf, fully expanded leaves were selected from eight-week-old plants. A minimum of three individual plants for each genotype were used as biological replicates. The total chlorophyll content was measured using a spectrophotometer (Hach DR6000, Loveland, CO, United States) at A665 and A648, following the protocol of Barnes et al. (1992).

### Measurement of Water Loss

The seedlings grown in a cell of 50-cell tray for 3 weeks were transplanted into pots (10 × 10 × 11 cm) containing the same amount of soil (300 g dry weight) and grown under constant conditions in a greenhouse. Irrigation and nutrients were supplied equally. The water loss of mature plants was measured using eight-week-old plants. At least three pots for each genotype were used as one experimental set. The potted plants were then placed in containers holding equal amounts of water. Empty pots containing soil were used as control. The initial total weight of the potted plant and water tank was measured. This was followed by periodic measurement of the weight and the greenhouse temperature and humidity every 2 h. The water was replenished every 2 days. The weight measured of each container was subtracted to the evaporation control during each period, then normalized against the final fresh weight and displayed as percentages. The water loss values indicate the percentage water loss per gram of fresh weight during each period. The experiment was performed over at least three consecutive days of cloudy and sunny conditions. The water loss assay for the aerial organs was performed using three-week-old seedlings with the roots removed. Five seedlings were used for quantification per genotype. The seedlings, sealed in Falcon tubes containing 50 ml of water, were placed into a growth chamber set at 25°C with a 16 h light (600w sodium lamps)/8 h dark cycle. The initial weight was measured, and the reduced weight was recorded daily in the evening for 4 days. Water loss was normalized against fresh weight and reported as a percentage.

### Measurement of Water Potential

Tillers at the heading and flowering stages were collected from mature plants and used to quantify water potential. The water potentials of the culms were measured using a Model 600 Pressure Chamber Instrument (PMS Instrument Company, Albany, OR, United States). The samples were prepared from at least six plants for each genotype. Three stems from each plant were collected for the measurement. The second node above the base of the culm was cut from the tiller before it was fixed to a fitting ring. The aerial parts of the plants were then placed in pressurized chambers. The water potential (Bar) was recorded as the nitrogen gas pressure when water escaped from the second node, and the reading was converted to megapascal (MPa).

### Photosynthetic Capacity Measurements

Fully expanded leaves were used for gas exchange measurements using a Licor 6400XT photosynthesis system (Licor Inc., Illinois, NE, United States). The measurements were taken at a block temperature maintained at 27°C, with a flow rate of 500 ml min^−1^, and 60–65% humidity. The CO_2_ concentration was 400 μmol mol^−1^ and the photosynthetically active radiation was 1,000 μmol·m^−2^·s^−1^ Each measurement took 2 min to achieve stability. The data contain stomatal conductance, transpiration rates, and photosynthetic activity. To minimize the effects of temporal changes in weather parameters, four genotypes (three plants per genotype) were grouped and used as a single set of measurements. The measurement was performed with the randomized order of these genotypes. Weather parameters (wind, humidity, temperature, daytime, and cloud cover) were considered for each measurement. The experiments were conducted more than three times on different days, and the most representative data were used. Chlorophyll fluorescence parameters were used to measure the photosynthetic capacity using a FluorPen FP 110 system (Photon System Instruments, Drásov, Czech Republic). Fully expanded leaves from nine-week-old plants were adapted for 30 min in the dark before their quantum yields were measured. Each genotype was represented by a minimum of 18 biological replicates (plants) were used. The quantum yields of photosystem II is equivalent to *Fv*/*Fm* in dark-adapted leaves, expressing the efficiency of photosystem II (Genty et al., 1989). The variable fluorescence of the dark-adapted leaves (*Fv*) was calculated from the *Fm − F0* relationship (Kalaji et al., 2014).

### Drought Resistance Assay

For the greenhouse-based drought resistance assay, five seeds were grown in each cell (5 × 5 × 5.5 cm) of a 50-cell tray under normal conditions. Each genotype was represented by 50 plants. The three-week-old seedlings were then subjected to drought stress by withholding water for 6 days. On the seventh day, the plants were re-watered, and photographs were taken 10 days after re-watering. The survival rate was calculated as the ratio of the number of surviving plants to the total number of plants. This experiment was repeated more than three times.

### Anatomical Characterization

The plant samples were fixed in 10% (v/v) formaldehyde, 5% (v/v) acetic acid, and 50% (v/v) ethanol and dehydrated in a graded ethanol series. For paraffin embedding, the ethanol series was followed by an ethanol/Histo-Clear II (National Diagnostics, Atlanta, GA, United States) series. The samples were finally embedded in paraffin (Paraplast X-tra; McCormick Scientific, St Louis, MO, United States), and sectioned at 10 μm with a HM 340E rotary microtome (Microm, Walldorf, Germany). The sections were stained with Toluidine Blue O (Sigma-Aldrich, St Louis, MO, United States) and observed under a light microscope (BX50; Olympus, Tokyo, Japan).

For scanning electron microscopy (SEM), the leaf and leaf sheath pulvini of three-week-old plants were immediately fixed in solution (2.5% glutaraldehyde and 0.1 M sodium cacodylate, pH 7.4) for 12 h, and then dehydrated in a graded ethanol series. The dehydrated samples of critical points were dried in liquid CO_2_ (Samdri^®^-795; Tousimis, Rockville, MD, United States) and mounted on metallic stubs (Electron Microscopy Sciences, Hatfield, PA, United States). The mounted specimens were coated with gold nanoparticles before viewing under the SEM (Philips XL30 SFEG; Philips, Eindhoven, Netherlands). For transmission electron microscopy, the vascular bundles of leaf sheath from three-week-old plants were fixed in 2% formaldehyde and embedded in low-viscosity (Spurr’s) resin (Electron Microscopy Sciences, Hatfield, PA, United States). Sections 85 nm thick were cut, post-stained with uranyl acetate and lead citrate, and observed using a Zeiss EM 902A transmission electron microscope (Carl Zeiss, Jena, Germany).

For internal 3D imaging of the xylem vessels using a high-resolution X-ray microscope (Xradia 620 Versa; Carl Zeiss), fully expanded leaf blades were sampled from eight-week-old plants. Midrib sections (5 × 2 × 1 mm, length × width × thickness) containing vascular bundles were dissected at the widest leaf blade width. The samples were dehydrated using a freeze dryer (Operon, Gimpo, South Korea) and mounted with epoxy glue on the head of an aluminum nail. They were then fixed and placed on the sample stage. The selected 3 Å–3 × 3 mm areas were X-rayed in a narrow section of the printed or mold-pressed samples. 3D images were obtained by rotating from −180° to 180° (total 360°), with a 40 keV acceleration voltage and 3 W power. The 3D tomographic image was reconstructed using the commercial software provided by Carl Zeiss.

Histochemical staining for GUS activity was performed by incubation in a 5-bromo-4-chloro-3-indolyl glucuronide solution (Kim et al., 2007). For the tissue-specific localization of *LPA1*, three-week-old seedlings of *LPA1-Ds/+* (*lpa1–2ds* heterozygotes) were collected in 15 ml Falcon tubes containing GUS-staining solution, as described previously (Chin et al., 1999). The samples were incubated at 37°C for 2 days in the dark and then dehydrated in a 30–70% graded ethanol series. To obtain tissue sections, the GUS-stained samples were dehydrated and embedded in paraffin, as described previously.

Stomata density was measured from fully expanded leaves using instant glue, as described previously (Kusumi, 2013). The samples were observed and captured under a light microscope (BX50; Olympus, Tokyo, Japan). Stomatal size was calculated using ImageJ v1.52.^1^ Typically, three to six plants per genotype and three measurements per leaf were examined, between the midrib and the margin area.

### Laser Capture and Microdissection

The shoot apices of three-week-old plants were cut and fixed in 100% acetone and were then hydrated in PBS solutions containing 10 and 15% sucrose for 12 h, respectively, and embedded *via* freezing. Sections 20 μm thick were cut from the embedded tissues with a SHANDON AS-620E cryotome (Thermo Electron Corporation, United States) and stained with Toluidine Blue O. Specific tissues were dissected out using a laser capture microscope (Leica, Bensheim, Germany). The collected samples were then used for RNA extraction using the Arcturus^™^ PicoPure^™^ RNA Isolation Kit (Thermo Fisher Scientific, Waltham, MA, United States) and RT-PCR. For RNA sequencing (RNA-seq), the immature vascular bundles were collected from the fifth leaf primordia (P5) by micro-hand dissection under the light microscope. Each biological replicate comprised a pool of at least 50 dissected vasculatures. Two or more biological replicates were used for RNA extraction using the Arcturus^™^ PicoPure^™^ RNA Isolation Kit (Thermo Fisher Scientific), followed by RNA-seq library preparation.

### RNA Extraction, RT-PCR, and qRT-PCR

Total cellular RNA was isolated using the RNeasy Plant Mini Kit (Qiagen, Valencia, CA, United States) and treated with on-column DNase (Qiagen), according to manufacturer’s protocols. A reverse transcriptase ReverTra Ace^®^ -α- (Toyobo, Osaka, Japan) transcription kit was used to synthesize the cDNA, according to the manufacturer’s instructions. For the semi-quantitative RT-PCR, *ACT1* mRNA (*ACTIN1*; McElroy et al., 1990) was used for normalization in quantifying the cDNA. The amplified PCR products were electrophoresed in 1.5% agarose gel, and gel-docs imaging was used for data recording. qRT-PCR was performed using iQ™ SYBR Green Supermix (Biorad, Hercules, CA, United States) and the qRT-PCR products were then quantified using the ΔΔCt formula. The values were normalized against *UBIQUITIN* and *ACT1*.

For the stem-loop qRT-PCR, the shoot apices of WT and *lpa1-3* were collected from at least 12 plants which were pooled in one falcon tube as one biological replicate. The collected samples were immediately frozen in liquid nitrogen. Total RNA was isolated using TRizol (Invitrogen, Frederick, MD, United States), according to the manufacturer’s protocols. The quantification of miRNA166 expression using three biological replicates was performed according to the protocol described by Teotia et al. (2017). The primers used in this study are listed in Supplementary Table S3.

### RNA-Seq and Data Analysis

For RNA-seq, total RNA of WT and *lpa1-3* was isolated using the Arcturus^™^ PicoPure^™^ RNA Isolation Kit (Thermo Fisher Scientific) and treated with on-column DNase (Qiagen), according to manufacturer’s protocols. The total RNA was analyzed for concentration and quality using an ND-1000 system (NanoDrop Technologies, Wilmington, DE, United States) and a 2100 Bioanalyzer (Agilent Technologies, Palo Alto, CA, United States). A total of 1 μg of RNA was used in library construction, with a Truseq Stranded mRNA Prep Kit (Illumina, Inc. San Diego, CA, United States), according to manufacturer’s protocol. Libraries from 70 to 370 bp (mean 150 bp) were constructed and sequenced using Illumina NovaSeq 6000 to generate 101 bp paired-end reads. The RNA-seq reads were deposited to the NCBI Sequence Read Archive under bioproject accession PRJNA722879.

Raw reads were checked for quality using FastQC v0.11.7^2^ and pre-processed to remove adaptor sequences and low-quality reads using Trimmomatic v0.36 (Bolger et al., 2014), with following parameters (ILLUMINACLIP: TruSeq3-PE-2.fa:2:30:10; LEADING: 20; TRAILING: 20; MINLEN: 25; phred33). A total of 461,991,196 raw reads were sequenced, and 438,101,122 (94.8%) clean reads were pre-processed. Of the clean reads, the Q30 (sequencing error rate < 0.1%) was over 99% and the GC content was approximately 54% for all libraries. The clean reads were aligned to rice reference gene sequences (IRGSP-1.0, version; 2019-06-26) using Bowtie v2.2.6 (Langmead et al., 2009) and the abundance of each transcript was estimated and normalized to transcript per kilobase million (TPM) values using RSEM v1.2.31 (Li and Dewey, 2011). To identify DEGs between WT and *lpa1-3*, the expression profiles were filtered using DESeq2 v1.26.0 (Love et al., 2014) with following parameters (sum of TPM values for all samples ≥4; 1 TPM/sample, fold change ≥1.5, and, value of *p* < 0.05). GO enrichment analysis of the DEGs was performed using the Fisher test and no correction in the GO database,^3^ and enriched GO terms were selected with value of *p* < 0.01.

### Statistical Analyses

Statistical analyses were performed with GraphPad Prism 8.0 (GraphPad Software, Inc., San Diego, CA, United States). Statistical analysis was carried out as described in the figure legends. The values of *p* denoted on the graphs were determined using two-tailed, Student’s *t*-test. Comparisons between groups were made using one-way ANOVA followed by Tukey’s correction for multiple comparisons. The different letters denote significant differences between groups at a value of *p* < 0.05. All data are expressed as mean ± SD.

## Results

### Water Use-Related Traits of *lpa1*

**Figure 1:**
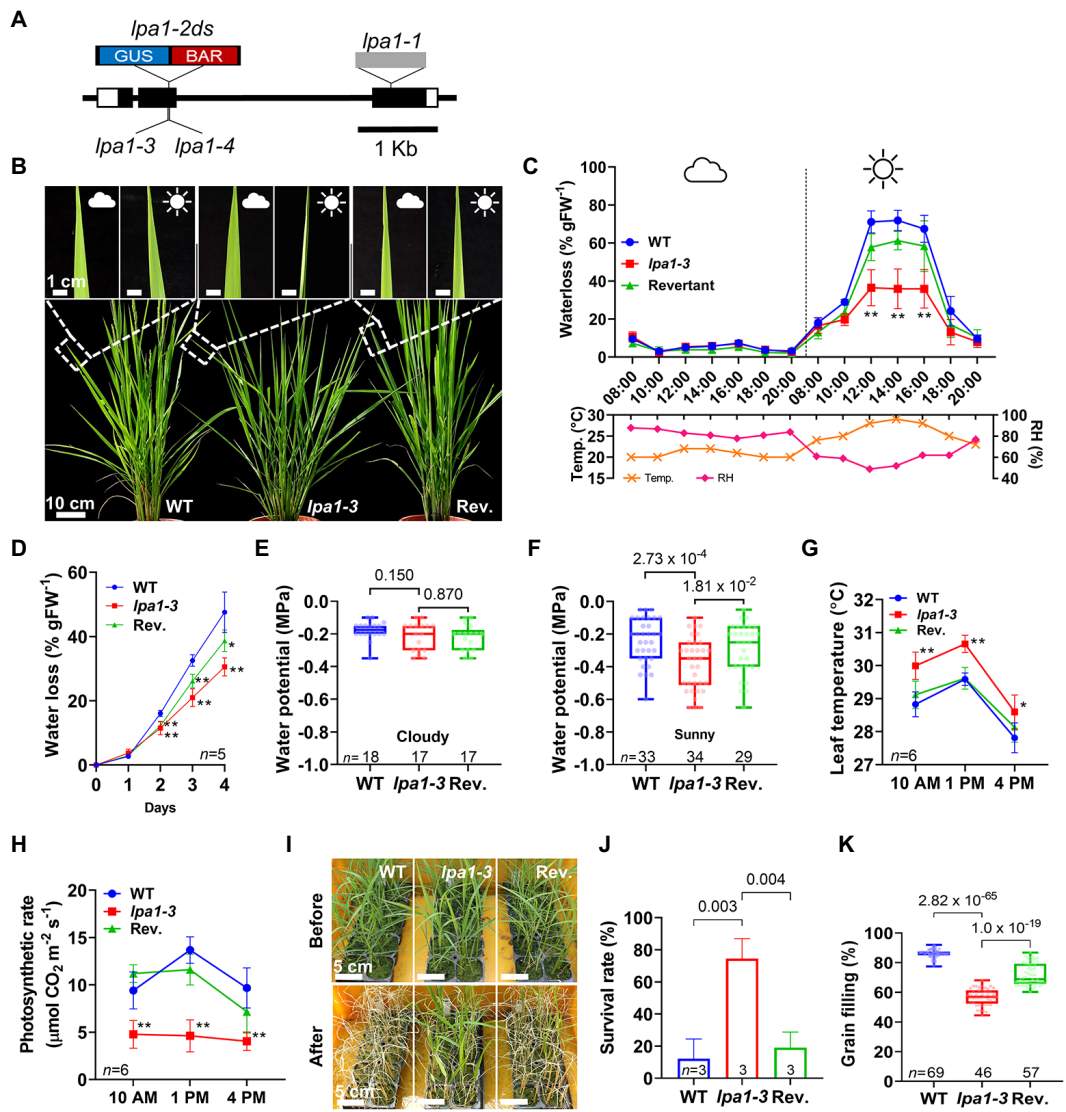
Water use-related traits of *lpa1*. **(A)** Genomic map of the locations of the *lpa1* alleles. Blue:red box and grey box indicate *Ds* and a *Ty1*-copia retrotransposon inserted in the exon (black box). GUS, *β-*glucuronidase; BAR, *basta*. **(B)** Representative field leaf phenotypes of wild type (WT), *lpa1-3*, and the revertant (Rev.). Inserts show the same leaves on cloudy and sunny days. **(C,D)** Quantification and comparison of water loss using whole plants in the vegetative stage on a cloudy and a sunny day **(C)**, and using the aerial organs of seedlings from which the roots were removed **(D)**, Temp., temperature; RH, relative humidity. Measured from five replicates for **(C)**. **(E–H)** Comparison of water potential under cloudy day **(E)** and under sunny day **(F)**, and leaf temperature **(G)** and photosynthetic rates **(H)** of WT, *lpa1-*3, and revertant at the three sequential time points (10 AM, 1 PM, 4 PM) on a sunny day. **(I)** Representative images of the three genotypes before and after subjected to drought stress. **(J,K)** Comparison of survival rates of the drought stress assay **(J)** and grain filling rate **(K)** of WT, *lpa1-3*, and revertant. Weather symbols indicate the local weather on a whole day. Bar and line graphs display mean ± SD. Values of *p* denoted on the graph, two-tailed, two-sample *t*-test (^*^p < 0.05; ^**^p < 0.01). For each box plot, the lower and upper bounds of the box indicate the first (Q1) and third (Q3) quartiles, respectively, the center line indicates the median. *n*, number of replicates.

In addition to identification of *lpa1-2ds* and revertant in our previous report, tissue cultures were used to generate transposon-free *lpa1* alleles by remobilizing the *Dissociation* (*Ds*) element at the *LPA1* locus. Notably, we isolated *lpa1-3* and *lpa1-4* with 7 and 2 bp insertions, respectively, at the *Ds* excision sites (Figure 1A).

In the field, all *lpa1* mutants showed leaf rolling on sunny days. However, the rolling phenotype was not detectable in the shade or on cloudy days (Figure 1B; Supplementary Figure S1). To understand the leaf rolling phenotype of *lpa1*, water losses on cloudy and sunny days were measured and compared among wild type (WT), *lpa1-3*, and revertant plants. The volume of water loss was significantly lower in *lpa1-3* than in WT or the revertant on sunny days. On cloudy days, there was no difference in water loss among the three genotypes (Figure 1C). Even after the roots were removed, the aerial parts of the mutants showed less water loss than those of WT or revertant (Figure 1D). To examine the force of water movement to the aerial tissues, we measured the water potentials of the stems on cloudy and sunny days. Compared to WT and revertant, *lpa1-3* showed normal water potential on cloudy days but significantly lower water potential on sunny days (Figures 1E,F). In addition, during leaf rolling, *lpa1-3* showed higher leaf temperatures than WT or revertant (Figure 1G; Supplementary Figure S2A).

In addition, transpiration rates and stomatal conductance were measured and compared among WT, *lpa1-3*, and revertant. On sunny days, the transpiration rates of *lpa1-3* were 39 and 48% lower than those of WT and revertant, respectively. However, the stomatal conductance was also 30–40% lower than those of WT and mutant (*lpa1-3* mean 0.07, WT mean 0.23 and revertant mean 0.17). Taken together, their trends were significantly lower in *lpa1-3* than in WT or the revertant. This indicated that *lpa1* suffered less evaporative water loss in the daytime (Supplementary Figures S2B,C). The photosynthesis rates were also calculated to evaluate water use in the leaves. The photosynthetic rate of *lpa1-3* was ~50% lower than those of WT and the revertant (mean value *lpa1-3* was 4.48 vs. 10.92 in WT and 9.99 in revertant; Figure 1H). However, their quantum yield values were similar, indicating that photosynthetic capacity is not altered in *lpa1-3* (Supplementary Figure S2D). The results indicated that *lpa1* has a low water content, which reduces water use on sunny days and subsequently evokes the leaf rolling phenotype. To examine whether low water use would confer drought tolerance in *lpa1* mutants, we conducted a recovery assay after imposing drought stress on plants. The data show significantly higher survival rates in *lpa1-3* than in WT or the revertant (Figures 1I,J). Therefore, the low water use of *lpa1* conveys resistance to drought stress.

We examined whether this low water use influenced grain filling during the reproductive stage, and lower grain filling rates per panicle were observed in *lpa1-3* (about 59%) compared to WT (84%) and the revertant (76%; Figure 1K). However, *lpa1-3* produced comparable numbers of tillers and spikelets to WT and the revertant (Supplementary Figures S2E–G).

### *LPA1* Controls Metaxylem Cell Size

**Figure 2:**
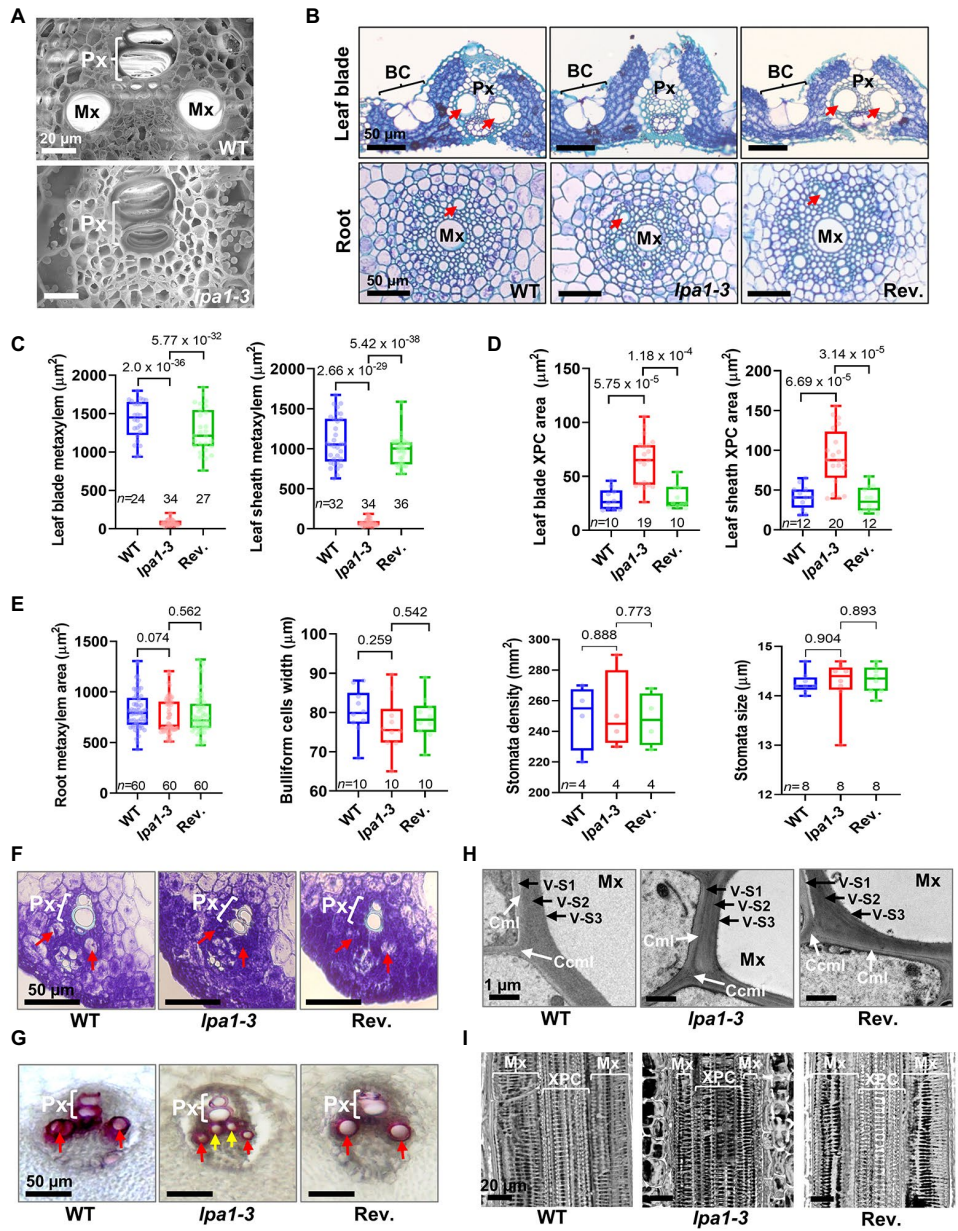
Anatomical analysis of metaxylems in leaf vasculatures of *lpa1-3*. **(A)** Scanning electron microscope images of vasculature in WT (top) and *lpa1-3* (bottom). **(B)** Cross sections of leaf blades (top) and roots (bottom) in WT, *lpa1-3*, and the revertant (Rev.). **(C,D)** Quantification and comparison of metaxylem (Mx) areas **(C)** and xylem parenchyma cell (XPC) areas **(D)** of leaf blades (left) and leaf sheaths (right). **(E)** Quantification and comparison of root metaxylem areas (left), bulliform cell (BC) widths (middle left), and stomata densities (middle right) and sizes (right). **(F)** Cross sections of vasculature in fifth leaf primordia to show the developing metaxylem. **(G)** Lignin staining of vasculature on the cross sections of pulvini tissues. **(H)** Transmission electron microscope images of secondary cell wall in mature metaxylem. White arrows indicate cell corner middle lamella (CCML) between adjoining fibers or compound middle lamella (CML). Mxv, metaxylem vessel; V-S1, outer secondary wall of vessel; V-S2, middle secondary wall of vessel; V-S3, inner secondary wall of vessel. **(I)** Internal 3D images of metaxylem and xylem parenchyma cells in leaf mid vein. Red arrows indicate metaxylem. Yellow arrows indicate xylem parenchyma cells. Px, protoxylem; For each box plot, the lower and upper bounds of the box indicate the first (Q1) and third (Q3) quartiles, respectively, the center line indicates the median. Values of *p* denoted on the graph, two-tailed, two-sample *t*-test. *n*, number of replicates. Scale bars are indicated in each panel.

Differences of water use between cloudy and sunny days suggested that *lpa1* may experience low water transport efficiency in aerial tissues. To inspect the morphological alterations in the vascular bundles, especially the xylem vessels, the vasculatures of the leaf sheaths of WT and mutants were examined using scanning electron microscopy (SEM). In *lpa1* mutants, enlarged metaxylems were absent, and protoxylems were normally developed (Figure 2A). All aerial parts (leaf blades, sheaths, and internodes) of mutants showed narrow metaxylems while metaxylems of the roots were normally enlarged (Figures 2B,C; Supplementary Figure S3). The diameters of the metaxylem vessels were significantly smaller in *lpa1-3* than in WT or revertant (Figure 2C). This was also observed for other *lpa1* alleles (Supplementary Figure S4). The dimensions of the xylem parenchyma cells in both the leaf blades and leaf sheaths were larger in *lpa1-3* than in WT or the revertant (Figure 2D). The morphologies and densities of bulliform cells and stomata showed no differences among the three genotypes (Figures 2B,E; Supplementary Figure S5). Thus, the absence of enlarged metaxylems was a major anatomical defect of the aerial organs of *lpa1*.

Metaxylem cells are mainly enlarged *via* the loosening of primary cell walls and plasma membranes, creating vessels equipped with thick secondary cell walls (Barros et al., 2015; Meents et al., 2018; Marzec-Schmidt et al., 2019). From histological sections of fifth leaf primordia, enlarged pre-metaxylem cells were detected in WT, but not in *lpa1-3* (Figure 2F). Lignification is an important process in secondary cell wall formation in xylem vessels (Zhong et al., 2011; Barros et al., 2015; Yoon et al., 2015). To further analyze secondary cell wall formation in the metaxylem, we performed vasculature lignin staining using pulvinal tissues with non-lignified parenchyma cells. In both the mutant and WT, lignin was detected in the secondary cell walls of the protoxylem and metaxylem (Figure 2G). However, the xylem parenchyma cells of *lpa1-3* were lignified earlier than those of WT. As shown in Figure 2G, *lpa1-3* had already accumulated lignin in the xylem parenchyma cells. The lignification of the primary xylem parenchyma cells might compensate for the smaller sizes of the metaxylem vessels in the water transport of *lpa1-3*. Using transmission electron microscopy, the layers and thickness of the secondary cell walls were examined. As in WT and the revertant, three layers (S1, S2, and S3) of secondary cell wall were detected in *lpa1-3*. There were no significant differences in the thickness of the cell walls among the three genotypes (Figure 2H).

To compare vessel structures between *lpa1-3* and WT, we performed 3D micro-CT imaging of vascular bundles in the leaf blades (Figure 2I; Supplementary Movies S1–S3). Similar annular and helical walls regularly arranged into protoxylem vessels were detected in *lpa1-3* and WT. The diameters of the cells were also similar in *lpa1-3* and WT. Moreover, the pitted or reticulate walls of the metaxylem vessels were similarly formed and patterned in WT, *lpa1-3*, and the revertant (Figure 2I; Supplementary Movies S1–S3). The longitudinally arranged walls were spaced at similar distances in *lpa1*, WT, and the revertant (Supplementary Movies S4–S6). Only the transversal circles of the walls were narrower in *lpa1-3* than in WT or the revertant. Thus, *lpa1* did not affect secondary cell wall formation or patterning in the xylem vessels. Vessel diameter was the only major morphological difference in the vasculature of *lpa1* and WT. The small vessel sizes in *lpa1* could be caused by xylem enlargement failure during primary cell wall expansion.

### *LPA1* Is Highly Expressed in Immature Xylem Cells

**Figure 3:**
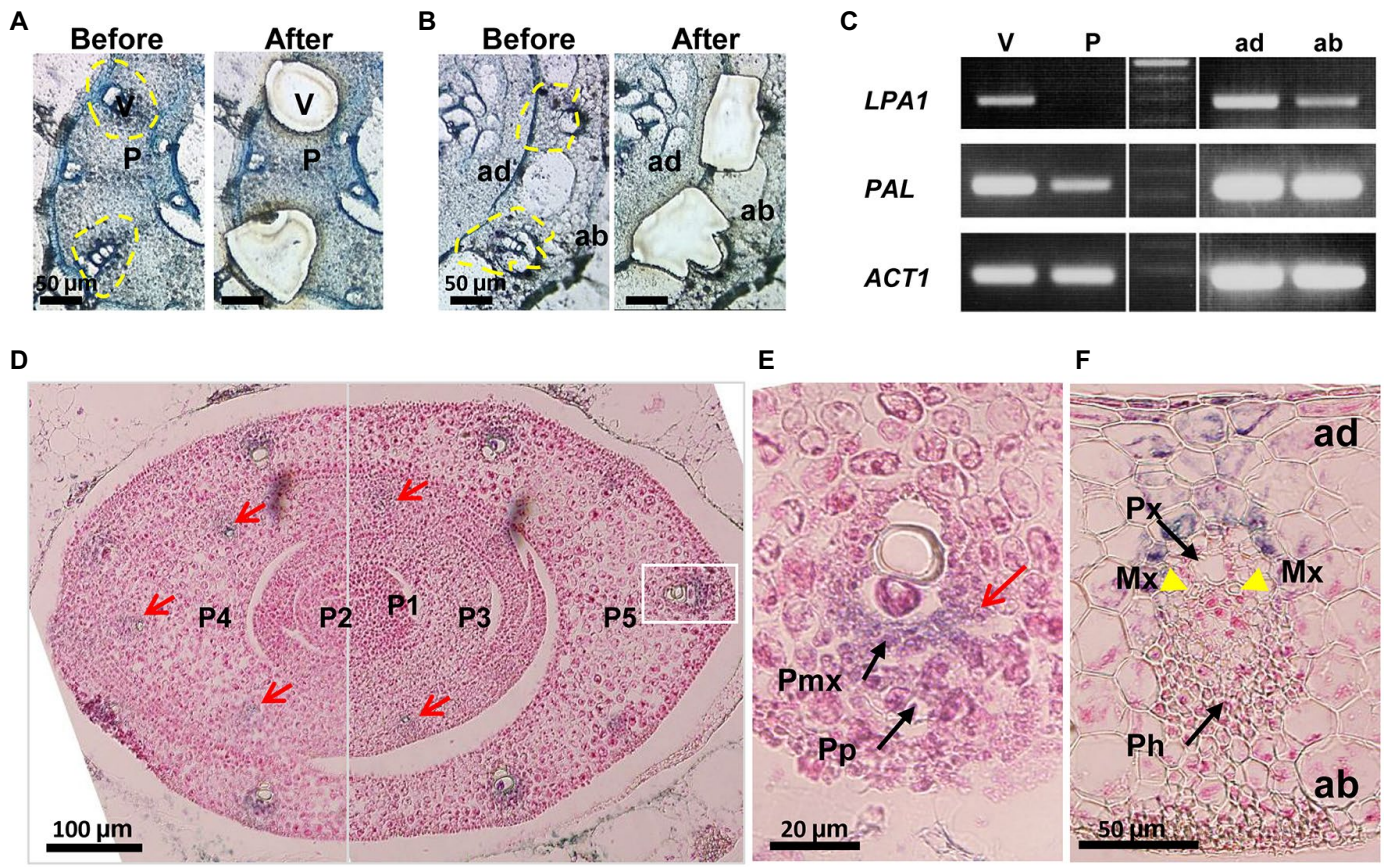
The expression patterns of *LPA1* in the vasculature of leaf primordia. **(A,B)** Cross sections of fifth leaf primordium **(A)** and leaf sheath pulvini **(B)** used for Laser Capture Microdissection (LCM). Left panel “Before” shows primordia before LCM while the right panel “After” shows those after LCM. V, vascular bundle; P, parenchyma tissue; ad, adaxial side leaf; ab, abaxial side leaf. **(C)** Semi-quantitative RT-PCR with cells collected by LCM. *Phenylalanine ammonia-lyase* (*PAL*) was used as a marker of vascular initiation and lignin accumulation. *Actin1* (*ACT1*) was used for normalization. **(D)** GUS expression in leaf primordia. Red arrows indicate GUS stains in the early vascular zones. P1–P5 indicate the leaf primordium number. **(E)** Section of vascular magnified white line box on the (d). GUS stains (red arrow) in vasculature at the stage of P5. Black arrows indicate pre-phloem (Pp) and pre-metaxylem (Pmx). **(F)** The absence of GUS expression (arrows) at the mature stage of vasculature. Yellow arrow heads indicate the enlarged metaxylem (Mx). Black arrows indicate protoxylem (Px) and phloem (Ph). Scale bars are shown in each panel.

*LPA1* expression was examined during the vascular development of leaf primordia and leaf sheath pulvini. The vasculature and parenchyma tissues of the fifth leaf primordia were collected using laser capture microdissection. Semi-quantitative RT-PCR analysis of the micro-dissected cells showed that *LPA1* was exclusively expressed in the pre-vascular bundle and was not expressed in the parenchyma cells of the fifth leaf primordia (Figures 3A–C). *LPA1* was highly expressed on the adaxial side, where the vascular bundles are located, and showed lower expression in the abaxial layers, which mostly consist of parenchyma cells (Figures 3A–C).

Three-week-old plants, heterozygous for the *LPA1*-*Ds* allele, were examined for *β*-glucuronidase (GUS) expression. The gene trap *Ds*, inserted at the *LPA1* locus, carried a GUS coding region for an *LPA1*-*GUS* fusion transcript (Figure 1A; Liu et al., 2016a). Histological staining showed that *LPA1* was expressed in the pre-vascular cells of the leaf primordia and the adaxial cells of the leaf sheath pulvini, but not in the mature vascular tissues (Figures 3D–F and Supplementary Figure S6). GUS staining was detected in the vascular initials in the ground tissues of the leaf and was relatively strong in the metaxylem area of the pre-vascular tissues, but it disappeared in the mature vascular tissues of the leaf sheath pulvini (Figures 3D–F and Supplementary Figure S6). Thus, *LPA1* expression was associated with cell wall enlargement during the xylem cell maturation stage of vascular bundle development.

### *LPA1* Acts on Cell Wall Formation in Immature Vasculature

**Figure 4:**
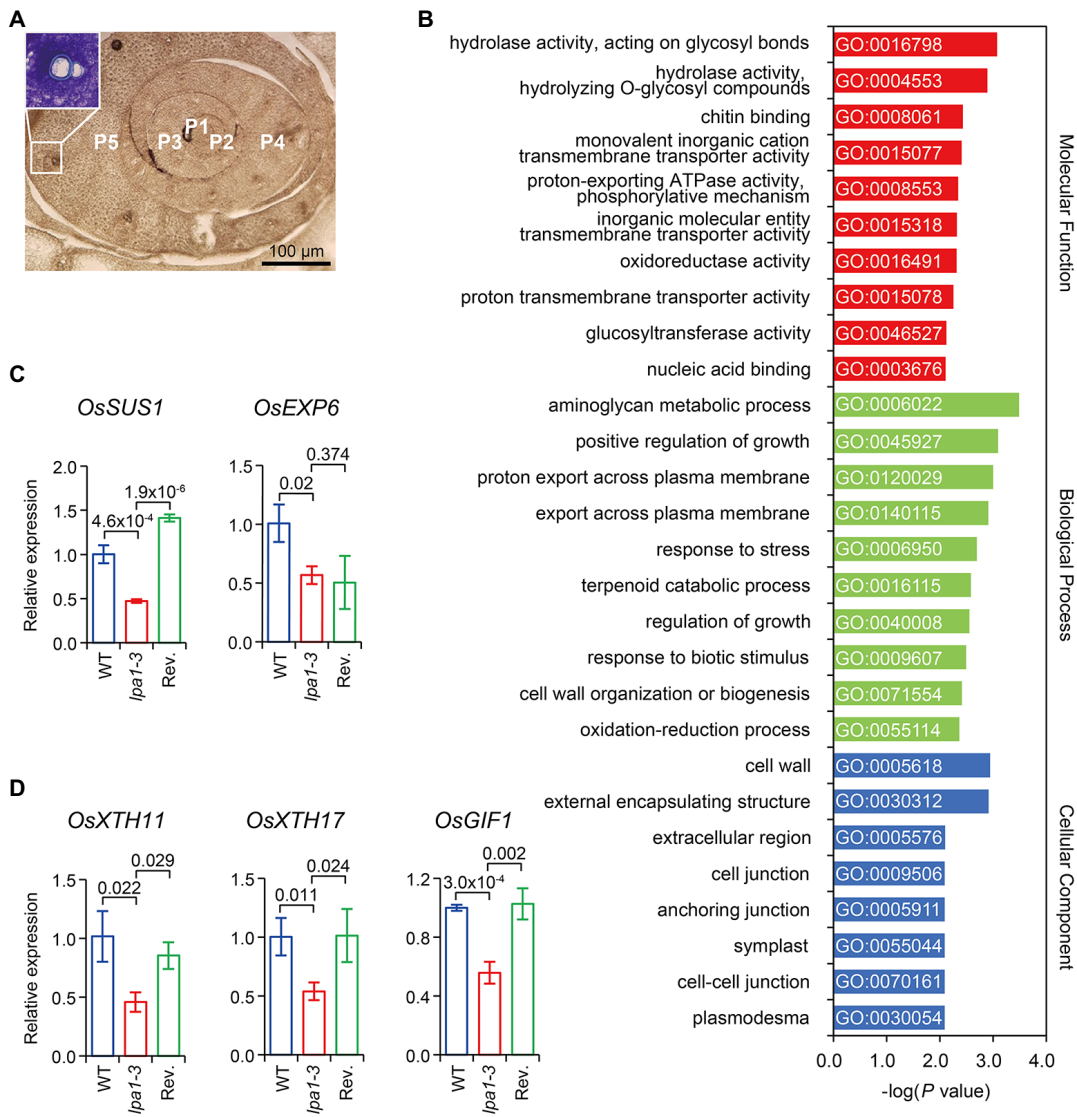
Many *LPA1*-dependent differentially expressed genes (DEGs) are related to cell wall organization. **(A)** Cross sections of immature vascular bundles from leaf primordia. The insert indicates the immature vascular bundles including protoxylems of transition stage (before enlargement of the metaxylem) in P5 that were micro-dissected for RNA-seq analysis. P1–P5 indicate the leaf primordium number. **(B)** Top 10 enriched gene ontology (GO) terms for each category in DEGs identified from WT and *lpa1-3*. **(C,D)** Expressions of genes related to cell wall synthesis, enlargement **(C)**, and cell wall loosening **(D)** in immature vascular. WT, blue line boxes; *lpa1-*3, red line boxes; revertant (Rev.), green line boxes. The expressions were determined by qRT-PCR relative to WT, normalized to *UBIQUITIN*. Bar graphs display mean ± SD. Values of *p* denoted on the graph, two-tailed, two-sample *t*-test. The immature vascular samples were dissected and collected from P5 at least 12 plants. Three biological replicates were used for qRT-PCR.

To understand the molecular roles of *LPA1* in metaxylem enlargement, we analyzed RNA-seq library transcriptomes from immature vascular bundles in the fifth leaf primordia of WT and *lpa1-3*. Data were captured using a modified microdissection method (Figure 4A). Comparison of WT and *lpa1-3* identified 223 differentially expressed genes (DEGs), with 90 upregulated genes and 133 downregulated genes (Supplementary Table S1). To investigate the biological roles of the genes regulated by *LPA1*, gene ontology (GO) enrichment analysis was performed on the DEGs in three GO categories (Supplementary Table S2). Most of the top 10 GO terms were related to cell wall organization, which involves the synthesis and remodeling of cell walls by exchanging cell wall components between the cytosol and plasma membrane (Figure 4B). Seven DEGs were selected from the GO assay for qRT-PCR analysis, to compare expression levels among WT, *lpa1-3*, and the revertant. *OsSUS1* and *OsEXP6*, which are related to cellulose biosynthesis and cell wall enlargement, were expressed at lower levels in *lpa1-3* than in WT (Figure 4C). *OsXTH11*, *OsXTH17*, and *OsGIF1*, which are related to cell wall loosening, were significantly downregulated in *lpa1-3* compared to WT and the revertant (Figure 4D). Notably, *OsGLN1;1* and *CHT4*, which are related to defense responses and cell wall loosening, were also downregulated in *lpa1-3* (Supplementary Figure S7). In contrast, the expression of miRNA166, which regulates five *OsHB* genes (Fouracre and Poethig, 2016; Zhang et al., 2018) and 15 genes related to vascular development and secondary cell wall formation (Schuetz et al., 2013), showed no significant differences between WT and *lpa1-3* (Supplementary Figure S8).

### Semi-Dwarf Growth of *lpa1* Improved Grain Filling and Maintained Drought Tolerance

**Figure 5:**
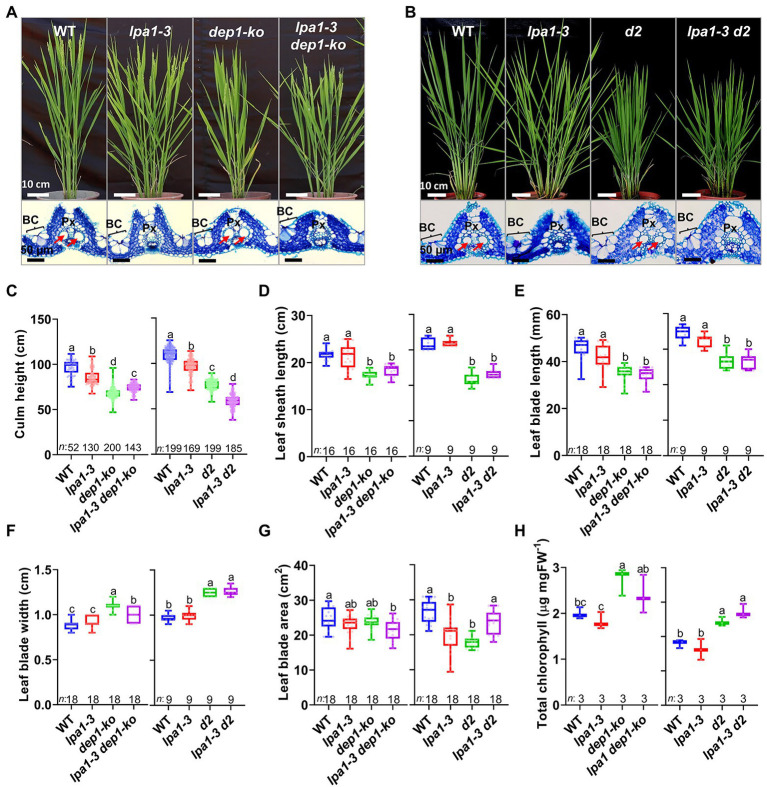
Field phenotypes, cross sections of vascular bundles, and morphological characteristics of *lpa1-3 dep1-ko* and *lpa1-3 d2*. **(A,B)** Representative plant growth and vascular bundles of WT, *lpa1-3, dep1-ko, lpa1-3 dep1-ko* **(A)**, and WT, *lpa1-3, d2, lpa1-3 d2* **(B)**. The lower section of the figures shows the cross sections of the leaf blades. Red arrows indicate the normally enlarged metaxylem vessels; BC, bulliform cells; Px, protoxylem. **(C–G)** Quantification and comparison of morphological characteristics which are culm height **(C)**, leaf sheath length **(D)**, leaf blade length **(E)**, leaf blade width **(F)**, and leaf blade area **(G)** among WT, *lpa1-3, dep1-ko, lpa1-3 dep1-ko* (left) and WT*, lpa1-3, d2, lpa1-3 d2* (right). **(H)** Quantification and comparison of total chlorophyll content in mature leaves among the eight genotypes. All the genotypes are BC2F3 generation which are the progenies of segregation after backcrossing three times. For each box plot, the lower and upper bounds of the box indicate the first (Q1) and third (Q3) quartiles, respectively, the center line indicates the median. The different letters denote significant differences between samples by one-way ANOVA followed by Tukey’s *post-hoc* test (*p* < 0.05). *n*, number of replicates. Scale bars are indicated in each panel.

Plant height determines gravity potential, which ultimately increases the hydraulic power for water supply to plants (Pratt et al., 2021). Therefore, the water potential of *lpa1-3* could potentially be increased by reducing plant height to provide sufficient water supply during plant growth and grain filling. Thus, *lpa1* was genetically crossed with *dense and erect panicle1-ko* (*dep1-ko*) and *ebisu dwarf* (*d2*), which produce semi-dwarf and erect phenotypes (Hong et al., 2003; Huang et al., 2009; Liu et al., 2021). In brief, *dep1-ko* is a T-DNA insertional mutant of *DEP1*, a γ subunit gene of the heterotrimeric G protein, showing semi-dwarf growth that is identical to that of *dep1* (Figure 5A). *d2* is a brassinosteroid-deficient mutant due to base substitution in *D2*, a CYP90D type of cytochrome P450 gene (Hong et al., 2003). Transverse leaf vasculature sections were prepared to inspect the sizes of the metaxylems. Neither of the double mutants had enlarged metaxylems; their metaxylem sizes were almost identical to those of *lpa1-3* (Figures 5A,B; Supplementary Figures S9A,B). However, the double genetic combinations, *lpa1-3 dep1-ko* and *lpa1-3 d2*, exhibited similar semi-dwarf traits to those of *dep1-ko* and *d2*. Compared to *lpa1-3*, the double mutants had shorter culm, leaf sheath, and leaf blade lengths (Figures 5C–E). Meanwhile, the leaf blade widths of the double mutants were wider than those of *lpa1-3*. Consequently, the total leaf blade areas were similar among all genotypes (Figures 5F,G). The chlorophyll content of the double mutants was much higher than that of *lpa1-3* and similar to that of *dep1-ko* and *d2* (Figure 5H). Moreover, the double mutants had similar numbers of large and small vessels to *dep1-ko* and *d2*, higher numbers than those found in *lpa1-3* and WT (Supplementary Figures S9C,D). Other plant architecture-determining factors in the double mutants, such as their tiller number and angles and leaf angles, showed similar average values to those of their parental mutants, resulting in the similar values to WT (Supplementary Figures S9E**–** G). Overall, *lpa1-3 dep1-ko* and *lpa1-3 d2*, which both contained non-enlarged xylem vessels, exhibited the semi-dwarf architecture phenotypes of *dep1-ko* and *d2*. In particular, the double mutants had wider leaf blades containing more small veins, a higher chlorophyll content, and relatively erect culms and leaves.

**Figure 6:**
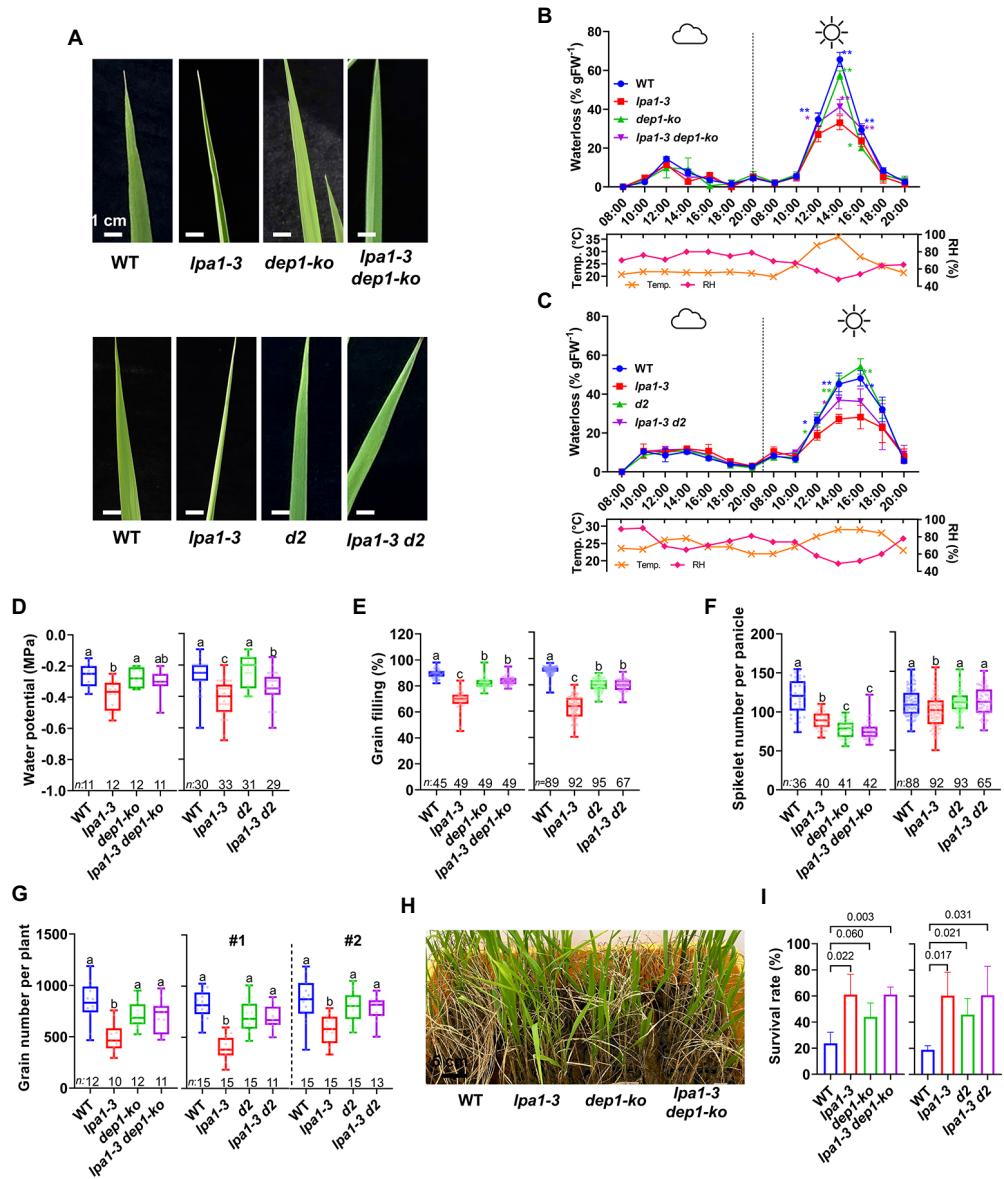
Water use-related traits, grain yield, and drought tolerance of *lpa1-3 dep1-ko* and *lpa1-3 d2*. **(A)** Representative leaf blade shapes among WT, *lpa1-3, dep1-ko, lpa1-3 dep1-ko* (top) and WT, *lpa1-3, d2, lpa1-3 d2* (bottom) on sunny days. **(B,C)** Water loss assay of WT, *lpa1-3, dep1-ko, lpa1-3 dep1-ko* **(B)** and WT, *lpa1-3, d2, lpa1-3 d2* **(C)** during cloudy and sunny conditions. Measured from a minimum of three replicates. ^*^p < 0.05; ^**^p < 0.01; *t*-test. Temp., temperature; RH, relative humidity. **(D–G)** Quantification and comparison of water potential on sunny days **(D)**, grain filling rates **(E)**, spikelet number per panicle **(F)**, and grain yield per plant **(G)** among WT, *lpa1-3, dep1-ko, lpa1-3 dep1-ko* (left) and WT, *lpa1-3, d2, lpa1-3 d2* (right). **(H)** Representative photographs of the surviving plants among WT, *lpa1-3, dep1-ko, lpa1-3 dep1-ko* after drought assay. **(I)** Survival rates of the drought resistance assay, quantified from three independent experiments, each consisting of 40–50 plants per genotype. All the genotypes are BC2F3 generation which are the progenies of segregation after backcrossing three times. Bar graphs display mean ± SD. For each box plot, the lower and upper bounds of the box indicate the first (Q1) and third (Q3) quartiles, respectively, the center line indicates the median. The different letters denote significant differences between samples by one-way ANOVA followed by Tukey’s post-hoc test (*p* < 0.05). *n*, number of replicates. Scale bars are indicated in each panel.

To examine whether these short culms containing narrow metaxylem vessels could efficiently transport water to the top of the plants on sunny days, we analyzed the water use characteristics of WT, *lpa1-3*, *dep1-ko*, *d2*, and their double mutants. Leaf rolling was no longer detected in either *lpa1-3 dep1-ko* or *lpa1-3 d2* on sunny days, despite their having narrow xylem vessels like *lpa1-3* (Figure 6A). The daytime water loss measurements showed that both double mutants significantly increased water use on sunny days, compared to *lpa1-3* (Figures 6B,C). The double mutants also showed a significant increase in water potential on sunny days (Figure 6D). The photosynthetic rate and other photosynthesis-related factors were also marginally recovered on sunny days (Supplementary Figure S10). However, the double mutants showed lower water use and photosynthetic efficiency than WT, *dep1-ko*, or *d2*. These results suggest that the partial recoveries in water use, and photosynthetic efficiency should be sufficient to release the double mutants from leaf rolling symptoms.

Since *lpa1-3* showed poor grain filling rates, we examined whether improvement of water use and photosynthesis in *lpa1-3 dep1-ko* and *lpa1-3 d2* could lead to the enhancement of grain yields. Single and double mutant lines, derived from the BC2F2, were grown in paddy field conditions during summer. *lpa1-3* produced about 74% of the spikelet number per panicle and 67% of the grain filling rate of WT plants (Figures 6E,F). In contrast, both *lpa1-3 dep1-ko* and *lpa1-3 d2* showed over 88% of the grain filling rates of WT. The spikelet numbers of the double mutants were also similar to those of *dep1-ko* and *d2* (Figures 6E,F). Two independent yield trials showed that the total grain number yield of *lpa1-3 d2* was comparable to that of *d2* and WT (Figure 6G). This study demonstrates that the yield potential of *lpa1-3 d2* was fully recovered and was similar to that of WT (Figure 6G).

Since *lpa1* is tolerant to drought stress, we examined whether *lpa1-3 dep1-ko* and *lpa1-3 d2* also exhibited drought tolerance during the vegetative growth stage. A drought recovering assay was performed with three-week-old seedlings. After 6 days of drought treatment, the plants were re-watered, and the survival rates were calculated. Interestingly, the double mutants showed comparable survival rates to *lpa1-3* (Figures 6H,I), exhibiting stronger drought tolerance than WT, *dep1-ko*, or *d2*. The semi-dwarf single mutants showed stronger drought tolerance than WT.

Overall, *lpa1* suppresses the enlargement of metaxylems, which results in low water transport efficiency and enhances drought tolerance. However, genetic combination with semi-dwarf genes complements the water transport efficiency, consequently leading to the recovery of grain yield. The double mutants also maintained strong drought tolerance.

## Discussion

To efficiently supply water to the plant body, the xylem differentiates from pre-vascular initials into protoxylem and metaxylem, which mainly enlarge during primary cell wall loosening and are equipped with thick secondary cell walls (Kubo et al., 2005; Spicer and Groover, 2010; Meents et al., 2018). *LPA1* is expressed in metaxylem tissues from the pre-vascular cell stage to the premature metaxylem stage in the leaf primordia but is not expressed in the mature vasculature (Figures 3D–F). The mutant *lpa1* alleles developed normal vascular structures, except that the small, narrow metaxylems failed to enlarge during the primary cell wall elongation stage. Interestingly, these narrow metaxylem were observed only in the aerial tissues, not in the roots. The transcriptome analysis indicates that *LPA1* regulates the expression of genes related to cell wall formation and elongation, such as *SUS1*, *EXP*, and *XTH* (Figures 4C,D; Cosgrove, 2000; Eckardt, 2008; Hirose et al., 2008; Maris et al., 2009; Hara et al., 2014). It is unlikely that *LPA1* is involved in xylem differentiation or the deposition of secondary cell walls, as the expression of the marker genes was not altered and there were no differences in xylem morphological characteristics or secondary cell wall structures between WT and *lpa1-3*. These results indicate that *lpa1* could take up and transport water *via* normal root xylem to aerial tissues with narrow metaxylems and normal protoxylems. Consequently, this restricted water supply system could result in the phenotypic differences in *lpa1* between sunny and cloudy days. Further work is required to understand why the protoxylem and root xylem are not altered in *lpa1*, even though *LPA1* is expressed in the protoxylem of leaf primordia and in the root vasculature. One might predict that certain interacting partners of *LPA1* show tissue-specificity.

Leaf rolling on sunny days is a typical response to drought conditions that relieves stress in the cells and bodies of plants. The leaves are usually rolled *via* structural variations in leaf cell types, such as bulliform, stomata, and xylem cells (Hsiao et al., 2008; Zhang et al., 2009; Liu et al., 2016b). Although *lpa1* has narrow metaxylem vessels, there are no morphological differences in bulliform cells, stomata, or other xylem vessels (including all root xylem vessels) between *lpa1* and WT (Figure 2E). Moreover, in *lpa1*, a water loss assay using aerial tissues without roots showed identical patterns to one using the whole plant (Figures 1C,D). These results indicate that *lpa1* has a normal transpiration system in the leaves and normal water transportation from the roots in the soil to the stem. Therefore, the rolling phenotype could result from limited water transportation from the stems to the leaves and a subsequent reduction of the water content, inducing this drought stress symptom (Zhang et al., 2018; Cal et al., 2019). This would explain why the leaves of *lpa1* rolled up on sunny days but not on cloudy days. In addition, limited water availability affects transpiration rate, stomatal conductance, and photosynthesis rate, which are all correlated with the availability of water vapor (Franks and Brodribb, 2005; Bertolino et al., 2019; Ramachandran et al., 2020). This results in significant decreases in the grain filling ratio and grain yield of *lpa1* (Figure 1K).

As crops now face periodic water shortages owing to climate change, rice breeding programs are searching for new genotypes that use water more efficiently under conditions of limited water availability (Wasson et al., 2012; Feng et al., 2016; Caine et al., 2019). *lpa1* could be a genetic candidate to enhance the efficiency of water use under water shortage or drought conditions, since *lpa1* developed narrow metaxylems that allowed the plants to survive strong drought stress. More importantly, *LPA1*-mediated narrowing of the metaxylems can be manifested in a semi-dwarf genetic background, such as *dep1* or *d2*. These genetic combinations successfully compensate for the negative morphological characteristics of *lpa1*, such as leaf rolling, low grain filling rates, and low grain numbers per panicle (Figure 6). Moreover, the wide tillers and leaf angles of *lpa1* were decreased in the double mutants, which is a positive characteristic that can enhance rice yields (Supplementary Figure S9). Compared to *lpa1*, the double mutants showed increased leaf width, leaf vein number, and chlorophyll content. Consequently, the photosynthetic potential was recovered in the double genetic combination. This study demonstrates that *LPA1* can be utilized to confer drought tolerance without any loss of plant growth or grain yield in dwarf rice.

In summary, our findings provide evidence that *LPA1* controls metaxylem enlargement in the aerial tissues, and influences water transport and drought tolerance. We also propose that the morphological combination of semi-dwarfs with narrow metaxylems can be utilized as a major crop breeding strategy to gain access to the efficient water use and drought tolerance. We suggest that, in major crops, such as wheat, the semi-dwarf variants that led to the green revolution could be further adapted to rapidly gain drought tolerance to optimize and maintain their yields through longer dry seasons, by creating targeted mutations on the orthologs of *LPA1*.

## Data Availability Statement

The datasets presented in this study can be found in online repositories. The names of the repository/repositories and accession number(s) can be found at: National Center for Biotechnology Information (NCBI) BioProject database under accession number PRJNA722879.

## Author Contributions

RP, JH, C-dH, and SP designed and planned the experiments, designed the research, and wrote the manuscripts. RP, JH, SK, SR, SY, D-HJ, CK, YHL, YKL, E-YC, TD, and SP performed the experiments. RP, JH, C-dH, and SP analyzed the data. SY, D-HJ, Y-KC, JB, YKL, E-YC, and TD provided technical assistance. All authors discussed the results and commented on the manuscript.

## Funding

This research was supported by “the National Research Foundation of Korea (NRF-2016R1C1B2015877, NRF-2017R1A4A1015594, and NRF-2020R1A2C1101915) funded by the Korean Ministry of Science, ICT and Future Planning” to SP. RP and SK were supported by a scholarship from BK21 plus program. RP and YKL were supported by “R&D Program of ‘Plasma Advanced Technology for Agriculture and Food (Plasma Farming)’ (EN2225)–9-1711124797” through the Korea Institute of Fusion Energy (KFE) funded by the Government funds, South Korea.

## Conflict of Interest

The authors declare that the research was conducted in the absence of any commercial or financial relationships that could be construed as a potential conflict of interest.

## Publisher’s Note

All claims expressed in this article are solely those of the authors and do not necessarily represent those of their affiliated organizations, or those of the publisher, the editors and the reviewers. Any product that may be evaluated in this article, or claim that may be made by its manufacturer, is not guaranteed or endorsed by the publisher.

## Footnotes

^1^ https://imagej.nih.gov/ij/

^2^ https://www.bioinformatics.babraham.ac.uk/projects/fastqc/

^3^ http://geneontology.org/
