# Supplementary material for: Narrow lpa1 Metaxylems Enhance Drought Tolerance and Optimize Water Use for Grain Filling in Dwarf Rice
Source: Front Plant Sci. 2022 May 10;13:894545. doi: 10.3389/fpls.2022.894545 (PMC9127761; doi:10.3389/fpls.2022.894545)
Supplement: Supplementary file 1 [file Data_Sheet_1.DOCX]

Supplementary Material

Article title: **Narrow lpa1 metaxylems enhance drought tolerance and optimize water use for grain filling in semi-dwarf rice**

Authors: Ryza A. Priatama^1,7,†^, Jung Heo^2,†^, Sunghoon Kim^1,9^, Sujeevan Rajendran^2^, Seoa Yoon^3^, Dong-Hoon Jeong^4^, Young-Kug Choo^1^, Jong Hyang Bae^3^, Chul Min Kim^3^, Youn Hee Lee^5^, Taku Demura^6^, Young Koung Lee^7^, Eunyoung Choi^8^, Chang-deok Han^1,*^, Soon Ju Park^2,*^

**List of Supplementary material**

**Fig. S1** **Leaf rolling phenotype in *lpa1* alleles on a sunny day**

**Fig. S2** ***lpa1* affects leaf temperature, transpiration rate, and stomatal conductance**

**Fig. S3** ***lpa1* alters the metaxylem size of the aerial organs**

**Fig. S4** **Metaxylem failed to enlarge in *lpa1-2ds* and *lpa1-1***

**Fig. S5** **Scanning electron microscopy (SEM) images of stomata**

**Fig. S6** **GUS expression in pulvini and roots of *lpa1-2ds***

**Fig. S7** **Expression of *OsGLN1;1* and *CHT4* related to cell wall loosening among WT, *lpa1-3* and the revertant**

**Fig. S8** **Expression of vascular development genes, miRNA166, and five *OsHB* genes**

**Fig. S9** **Cross sections of vascular bundles and morphological characteristics of *lpa1-3 dep1-ko* and *lpa1 d2***

**Fig. S10** **Water-use characteristics of *lpa1-3 dep1-ko* and *lpa1-3 d2* double mutants**

**Table S1 DEG profiles between WT and *lpa1-3*.**

**Table S2 Enriched GO terms of DEGs between WT and *lpa1-3***

**Table S3 Primer sequences used for this study**

[All supplementary table is provided in a single MS Excel (.xls) file format].

**Video/Movie S1 The 3D micro-CT imaging** **video of WT metaxylem**

**Video/Movie S2 The 3D micro-CT imaging** **video of *lpa1* metaxylem**

**Video/Movie S3 The 3D micro-CT imaging** **video of revertant metaxylem**

**Video/Movie S4 The 3D micro-CT imaging** **video of the closeup at the metaxylem area in WT**

**Video/Movie S5 The 3D micro-CT imaging** **video of the closeup at the metaxylem area in *lpa1***

**Video/Movie S6 The 3D micro-CT imaging** **video of the closeup at the metaxylem area in revertant**


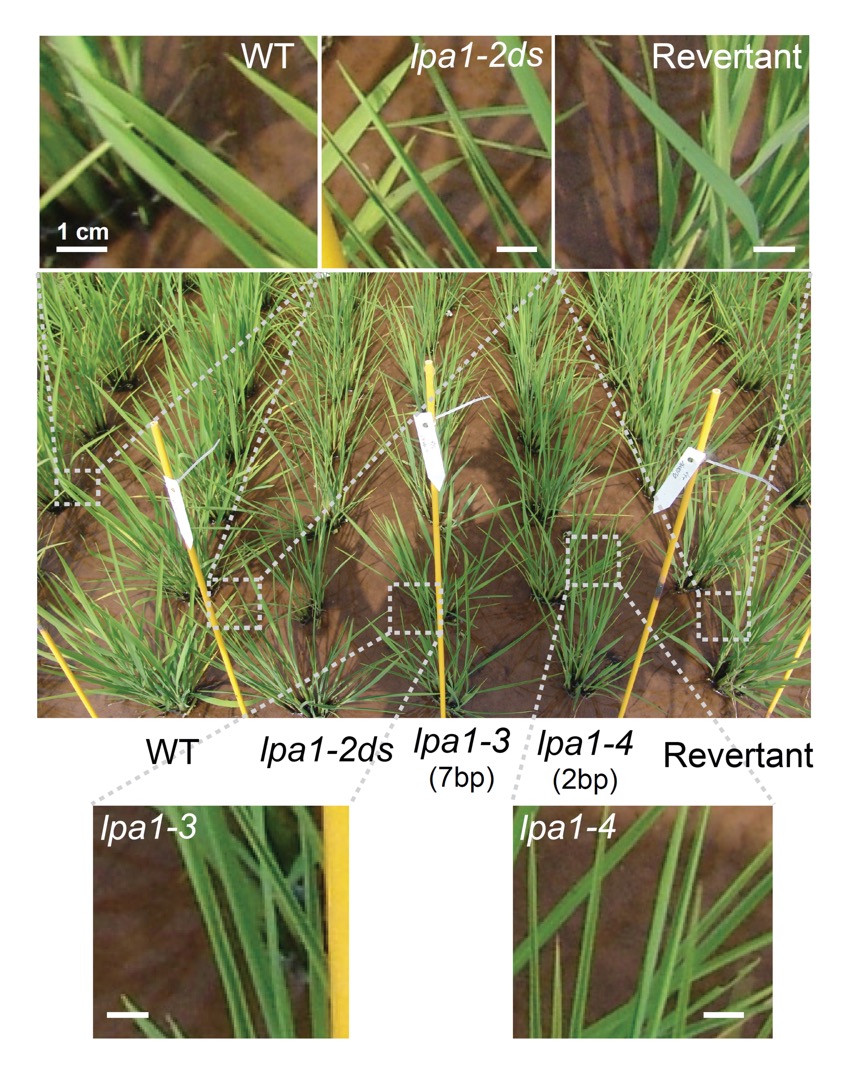


**Figure S1. Leaf rolling phenotype in *lpa1* alleles on a sunny day.**

Representative leaf phenotype from seven-weeks-old plants of wild type (WT), *lpa1-2ds, lpa1-3, lpa1-4,* and the revertant on a sunny day in a paddy field. Leaf rolling phenotypes were compared using magnified images of WT, *lpa1-2ds,* and the revertant (top), and *lpa1-3,* and *lpa1-4* (bottom). Dashed lines and boxes indicate the original positions of the magnified images. Scale bar, 1 cm.

**
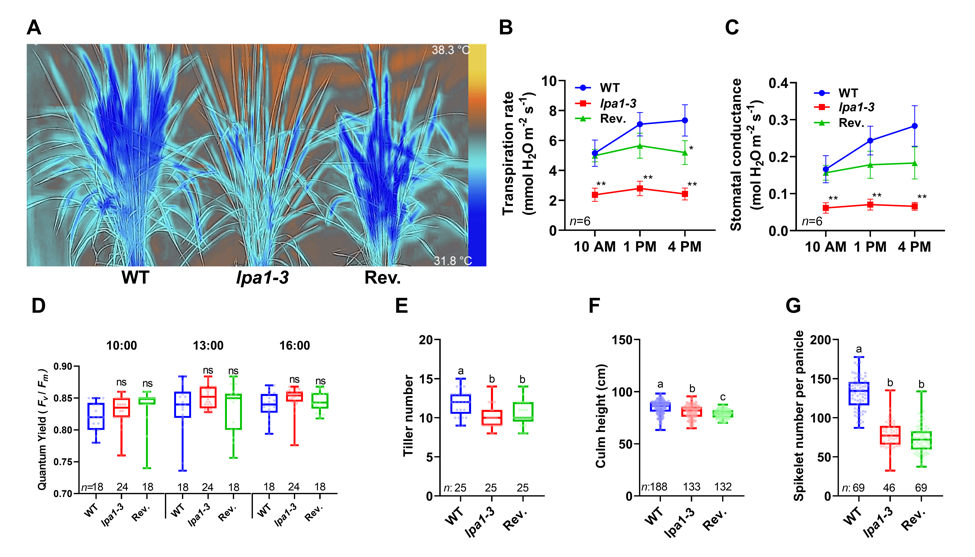
**

**Figure S2. *lpa1* affects leaf temperature, transpiration rate, and stomatal conductance.**

**(A)** Thermal camera imaging of the wild type (WT), *lpa1*, and the revertant on sunny days. **(B–D)** Quantification and comparisons of transpiration rate **(B)**, stomatal conductance **(C)**, photosynthetic capacity **(D)** among WT, *lpa1-3* and the revertant at the three sequential time points (10 AM, 1 PM, 4 PM) on a sunny day. **(E–G)** Quantification and comparisons of tiller number **(E)**, stem length **(F)**, spikelet number per panicle **(G)** among WT, *lpa1-3* and the revertant. Line graphs display mean ± SD. *P* values denoted on the graph, two-tailed, two-sample *t*-test. The different letters denote significant differences between samples by one-way ANOVA followed by Tukey’s post-hoc test (*P* < 0.05).


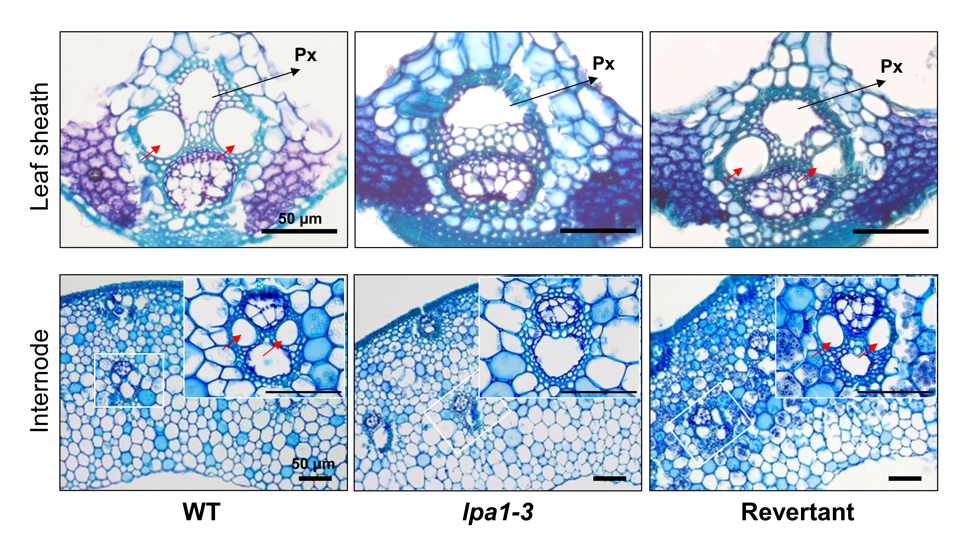


**Figure S3. *lpa1* alters the metaxylem size of the aerial organs.**

Cross sections of leaf sheath (top) and internode of culm (bottom) in WT, *lpa1-3* and the revertant. Inserts show magnified vascular bundles. Red arrows indicate the normally enlarged metaxylem. Black arrows indicate protoxylem (Px). Scale bars are shown in each panel.

**
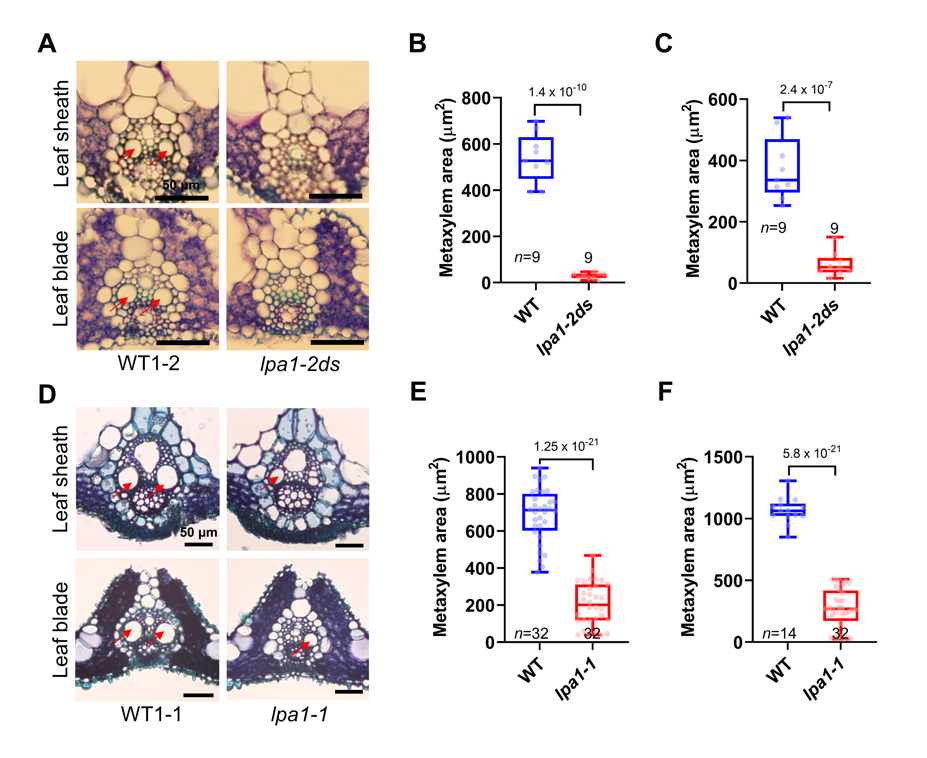
**

**Figure S4. Metaxylem failed to enlarge in *lpa1-2ds* and *lpa1-1*.**

**(A)** Cross section of leaf blade and leaf sheath of *lpa1-2ds* and its segregated WT. Red arrows indicate the normally enlarged metaxylem. **(B and C)** Quantification and comparison of metaxylem area in the leaf blade **(B)** and leaf sheath **(C)**, respectively. **(D)** Cross section of the leaf blade and leaf sheath of *lpa1-1* and its segregated WT. Red arrows indicate the normally enlarged metaxylem. **(E and F)** Quantification of metaxylem area in the leaf blade **(E)** and leaf sheath **(F)**, respectively. For each box plot, the lower and upper bounds of the box indicate the first (Q1) and third (Q3) quartiles, respectively, the center line indicates the median, and the whiskers represent data range, bounded to 1.5 * (Q3–Q1). *P* value denoted on the graph, two-tailed, two-sample *t*-test. Scale bars are indicated in each panel.

**
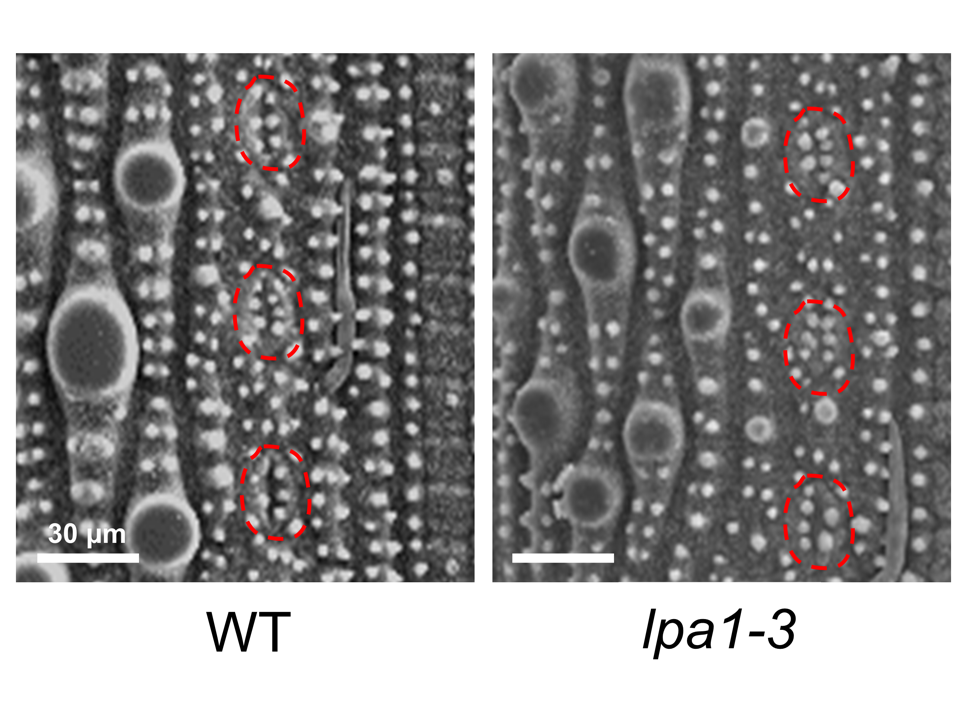
**

**Figure S5.** **Scanning electron microscopy (SEM) images of stomata.**

SEM captured the surface of abaxial side in mature leaf of WT (left) and *lpa1-3* (right). Red dotted circles indicate stomata. Scale bar 100 µm.

**
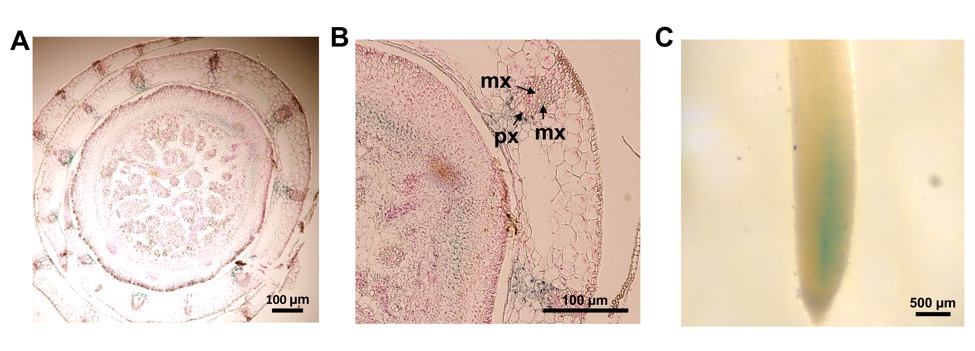
**

**Figure S6. GUS expression in pulvini and roots of *lpa1-2ds*.**

**(A)** Cross section of the basal pulvinus and GUS expression in the node and adaxial side of leaf sheath. **(B)** GUS expression of LPA1 in the adaxial layer of the pulvini but not in the mature vasculature. Black arrows indicate protoxylem (px) and metaxylem (mx). **(C)** GUS expression of LPA1 in root of three-weeks-old seedling.

**
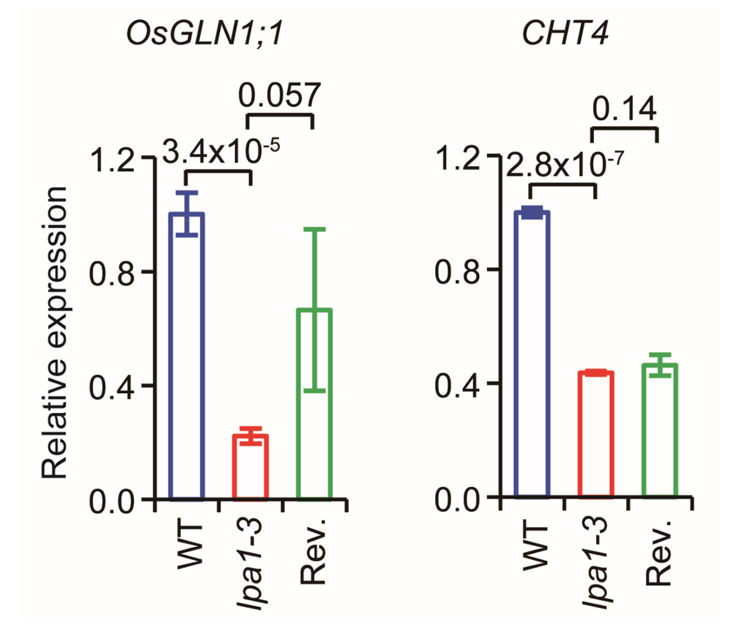
**

**Figure S7. Expression of *OsGLN1;1* and *CHT4* related to cell wall loosening among WT, *lpa1-3* and the revertant.**

The expressions of *OsGLN1;1* (left) and *CHT4* (right) in immature vascular were determined by qRT-PCR relative to wild type (WT, blue line boxes), normalized to *UBIQUITIN*. Red line boxes and green line boxes indicate *lpa1-3* and the revertant, respectively. Bar graphs display mean ± SD. *P* values denoted on the graph, two-tailed, two-sample *t*-test. Three biological replicates were used for this qRT-PCR assay.

**
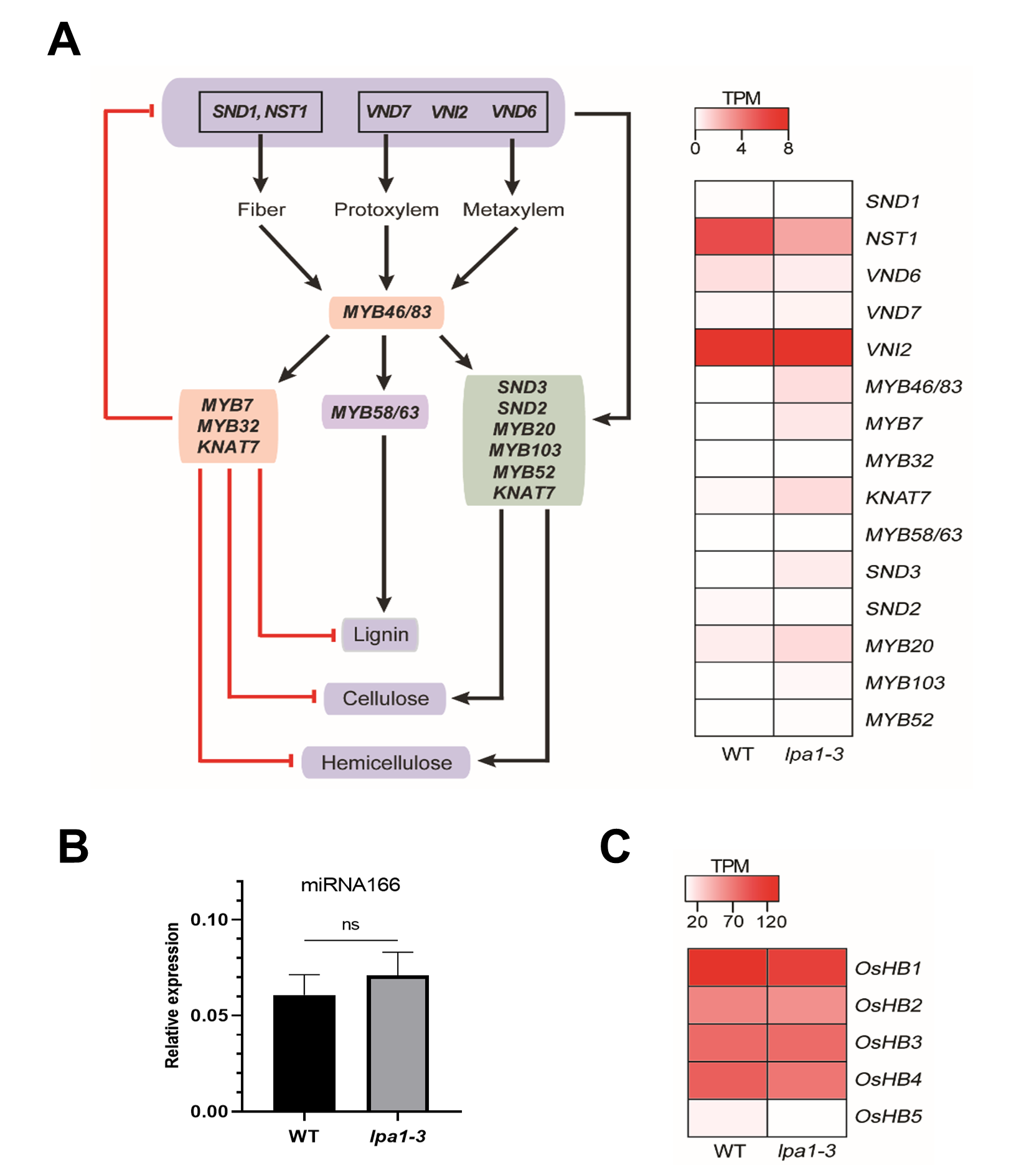
**

**Figure S8. Expression of vascular development genes, miRNA166, and five *OsHB* genes.**

**(A)** Overview of the functional relationships among genes involved in secondary cell wall formation and the expression of these genes in WT and *lpa1-3*. The genetic network of xylem development and secondary cell wall formation was modified from Schuetz et al. (2013)^1^. **(B)** Expression of miRNA166 using stem-loop qRT-PCR. The expression was determined by qRT-PCR relative to the *EF1α* gene. Box plots display mean ± SD. *P* value denoted on the graph, two-tailed, two-sample *t*-test. Three replicates were used for this qRT-PCR. **(C)** Expression of five *OsHB* genes regulated by miRNA166. In **(A)** and **(C),** normalized expression (TPM, transcripts per kilobase million) for WT and *lpa1-3* in protoxylems at the transition stage (fifth leaf primordium).

**
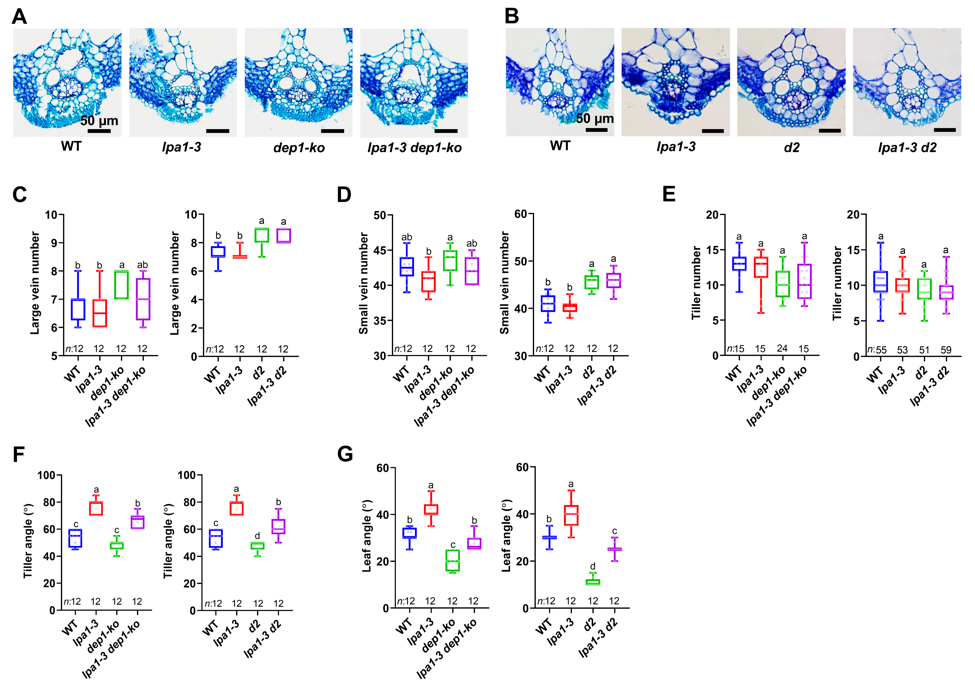
**

**Figure S9. Cross sections of vascular bundles and morphological characteristics of *lpa1-3 dep1-ko* and *lpa1 d2*.**

**(A and B)** Cross section of the leaf blades of WT*, lpa1-3, dep1-ko, lpa1-3 dep1-ko* **(A)** and WT, *lpa1-3, d2, lpa1-3 d2* **(B)**. **(C–G)** Quantification of morphological characteristics; numbers of large and small veins in leaf blades **(C and D)**, tiller number **(E)**, tiller and leaf angles **(F and G)** of WT*, lpa1-3, dep1-ko, lpa1-3 dep1-ko* (left) and WT, *lpa1-3, d2, lpa1-3 d2* (right)*.* All genotypes are BC2F3 generation which are the progenies of segregation after backcrossing three times. For each box plot, the lower and upper bounds of the box indicate the first (Q1) and third (Q3) quartiles, respectively, the center line indicates the median, and the whiskers represent data range, bounded to 1.5 * (Q3–Q1). The different letters denote significant differences between samples by one-way ANOVA followed by Tukey’s post-hoc test (*P* < 0.05). *n*, number of replicates. Scale bars are indicated in each panel.

**
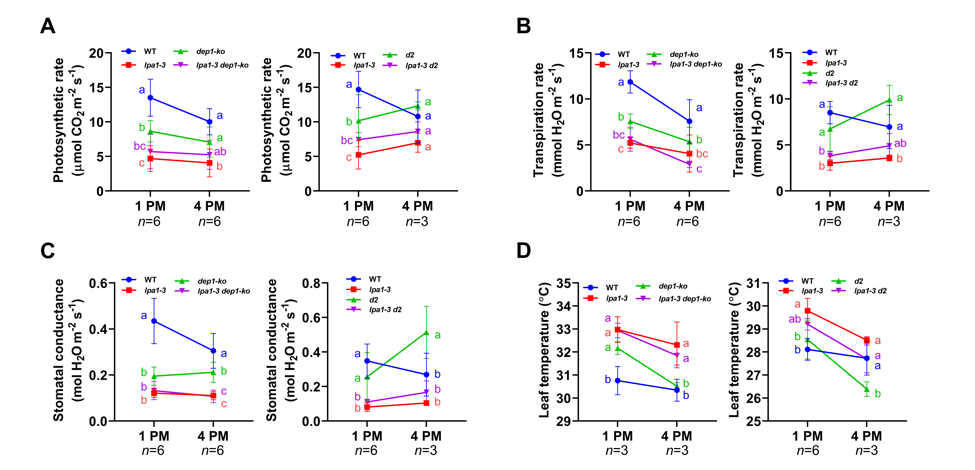
**

**Figure S10. Water-use characteristics of *lpa1-3 dep1-ko* and *lpa1-3 d2* double mutants.**

**(A–D)** Quantification of water-use characteristics which are photosynthetic rate **(A)**, transpiration rate **(B)**, stomatal conductance **(C)**, and leaf temperature **(D)** of WT*, lpa1-3, dep1-ko, lpa1-3 dep1-ko* (left) and WT, *lpa1-3, d2, lpa1-3 d2* (right). All genotypes are BC2F3 generation which are the progenies of segregation after backcrossing three times. Line graphs display mean ± SD. *n*, number of replicates. The different letters denote significant differences between samples by one-way ANOVA followed by Tukey’s post-hoc test (*P* < 0.05). *n*, number of replicates.
